# Ecofriendly Elimination of Ni (II) Using Fabricated Nanocomposite Based on Chitosan/Silver Nanoparticles/Carbon Nanotubes

**DOI:** 10.3390/polym15132759

**Published:** 2023-06-21

**Authors:** Eid M. S. Azzam, Walaa I. Elsofany, Fahad Abdulaziz, Hind A. AlGhamdi, Abdullah Y. AL alhareth

**Affiliations:** 1 Department of Chemistry, College of Science, University of Ha’il, Ha’il 81451, Saudi Arabia; wa.ibrahim@uoh.edu.sa (W.I.E.); fah.alanazi@uoh.edu.sa (F.A.); abdulla_medshil@hotmail.com (A.Y.A.a.); 2 Egyptian Petroleum Research Institute, Cairo 11727, Egypt; 3 Photochemistry Department, Chemical Industries Research Division, National Research Centre, 33 EL Buhouth St., Dokki, Giza 12622, Egypt; 4 Chemistry Department, College of Science, Imam Abdulrahman Bin Faisal University, Dammam 31441, Saudi Arabia; hasalghamdi@iau.edu.sa

**Keywords:** biopolymer, silver nanoparticles, carbon nanotubes, ecofriendly nanocomposites, wastewater, Ni (II)

## Abstract

Nickel ions are hazardous heavy metals that are non-biodegradable and can lead to allergic sensitivity and dermatitis. Nanomaterials are chosen for their effective elimination of impurities from water structures based entirely on the variety of therapy and degree of purification. The target of this work was the combination of the properties of biopolymers such as chitosan, silver nanoparticles (SNPs), and carbon nanotubes (CNTs) in one ecofriendly compound for Ni (II) uptake from the aqueous solution. To attain this target, the endeavor was made by creating a nanocomposite based on chitosan/SNPs/CNTs. The characterization of the structure of the fabricated nanocomposite (Chit-SNPs-CNTs) was carried out using different techniques. The removal of Ni (II) was examined by studying the adsorption of Ni (II) ions onto the fabricated nanocomposite by batch adsorption using UV, XRD, XPS, and ICP techniques. Moreover, we investigated the effect of the contact time, pH of the solution, and mass of the adsorbent on the efficiency of the adsorption of Ni (II). The results show that the adsorption capacity of Ni (II) increased by increasing the contact time with a neutral pH. The maximum removal of Ni (II) ions (99.70%) was found using 0.3 g of the (Chit-SNPs-CNTs) nanocomposite. In addition, the results indicate that the fabricated nanocomposite has a high adsorption effectivity, which is associated to the function of the chitosan, SNPs, and CNTs in upgrading the adsorption efficiency. Finally, the results in the existing work indicate that the ecofriendly nanocomposite organized here gave excessive effectivity closer to the elimination of Ni (II).

## 1. Introduction

Water is an integral but restricted useful resource that all dwelling matters want to access. The viability of clean water is the most necessary factor for life on the earth. Because of the intense pressure introduced by the increasing human population, industrialization, civilization, environmental changes, and agricultural activities, water shortage is a world problem that impacts even water-rich regions. The difficulty in obtaining the right of entry to smooth water is unavoidable, and will take widespread learning about how to increase new, much-less-high-priced strategies of purification while considering electricity utilization and environmental effects [1]. Water pollution consists of all kinds of liquid pollution, such as river and ocean contamination. Liquid pollution, as the phrase implies, occurs in quick liquid-containing regions, such as bays, streams, rivers, lakes, and subterranean water. It entails the discharge of toxic materials, pathogenic microorganisms, substances that want a large amount of oxygen to break down, and substances that are without problems regarding solubility, radiation, etc., that are deposited on the back side and whose accumulations will influence the fitness of aquatic ecosystems. Heavy metals such as lead (Pb), arsenic (As), mercury (Hg), chromium (Cr), especially hexavalent chromium, nickel (Ni), barium (Ba), cadmium (Cd), cobalt (Co), selenium (Se), and vanadium (V), as well as oils and grease, pesticides, etc., are some of the pollutions that are extraordinarily harmful, toxic, and toxic even in the ppb (parts per billion) range [2]. Nickel ions are hazardous heavy metals that are non-biodegradable and can lead to allergic sensitivity and dermatitis. The highest approved amount of Ni in US effluents from electroplating procedure wastewater is 4.1 mg/L, whereas that in ingesting water needs to be much less than 0.1 mg/L, in accordance with World Health Organization rules. Industrial tactics along with electroplating, battery production, mining, steel polishing, and forging are the major producers of nickel air pollution in water [3]. The desalination of sea and brackish water; extensive reuse of wastewater; disinfection and decontamination of water, i.e., biosorption and nanoadsorption for contaminant removal; nanophoto catalysis for the chemical degradation of contaminants; nanosensors for contaminant detection; and unique membrane-utilized sciences along with reverse osmosis, nanofiltration, ultrafiltration, and electrodialysis are all examples of nanotechnology applications in water treatment that are being developed and used [1]. Based on their physical and surface characteristics, nanomaterials are chiefly divided into countless classes. Carbon nanotubes (CNTs), metallic nano-adsorbents (Al_2_O_3_, ZNO, TiO_2_, and CeO_2_ nanoparticles), metal nanoparticles (Au and Ag), combined oxide nanoparticles (Fe-Ti nanoparticles), polymer nano-adsorbents, nanofibers, and nanoclays are examples of nanomaterials. In addition, they make use of nanoscale holes determined in zeolite filtration membranes and nanocatalysts [4].

Three-dimensional (3D) carbon nanomaterial assemblies are of great interest in emerging applications [5]. Carbon nanotubes (CNTs), a novel adsorbent that was first developed in 1991 by using Iijima, have been tested to currently be of greater quality than activated carbon. In the literature, there are, however, distinctly few investigations on the absorption of heavy metals with CNTs [6]. The extremely good adsorption conducts carbon nanotubes (CNTs) toward several hazardous contaminants. These adsorbents are very fine in casting off unique pollution due to their excessive adsorption effectivity and high adsorption rate. These traits make CNTs the present day substance for the purification of water contaminated with heavy steel ions and dyes [4]. The most time-honored biopolymer after cellulose is chitosan. It has been utilized in water purification systems for over thirty years. In recent years, a new type of unique polymeric material with intrinsic microporosity (PIM) has emerged as a practical material for a range of technologies, such as membrane separation, catalysis, energy storage, sensors, and so on [7]. Chitosan, a polysaccharide that takes place naturally and is located in crabs, shrimps, lobsters, coral jellyfish, butterflies, ladybugs, and one-of-a-kind creatures, is the deacetylated structure of chitin. Because it possesses free amino groups, chitosan is a higher first-rate chelating agent than chitin [8]. Since chitosan has free amino groups, it can chelate substances more effectively than chitin. Biocompatibility, biodegradability, and an improved medication absorption are among chitosan’s unique properties. Chitosan should therefore be viewed as a young polymer that is useful for analytical chemistry and water treatment [8]. For wastewater treatment, chitosan (clay-containing silver nanoparticles)-based nanocomposites were utilized [8]. However, to enhance the absorption potential and separation rate, the sketch and synthesis of novel adsorbents relying on chitosan nanocomposites are still needed [8]. In the present work, we fabricated an ecofriendly and simple nanocomposite based on chitosan, CNTs, and silver nanoparticles (SNPs) for the uptake of Ni (II) ions from wastewater. We investigated the sorption of Ni (II) ions using different techniques, such as FTIR, UV-Vis, EDX, XRD, XPS, SEM, TEM, ICPE, and atomic adsorption. The sorption of Ni (II) ions was studied in this work by a variation in different parameters, such as the contact time, pH of the solution, and mass of the fabricated nanocomposite (Chit-CNTs-SNPs) as the absorbent.

## 2. Materials

All of the chemicals used in this project came from the German Sigma-Aldrich company (St. Louis, MO, USA).

### 2.1. Synthesis of Chitosan from Chitin

**Scheme 1:**
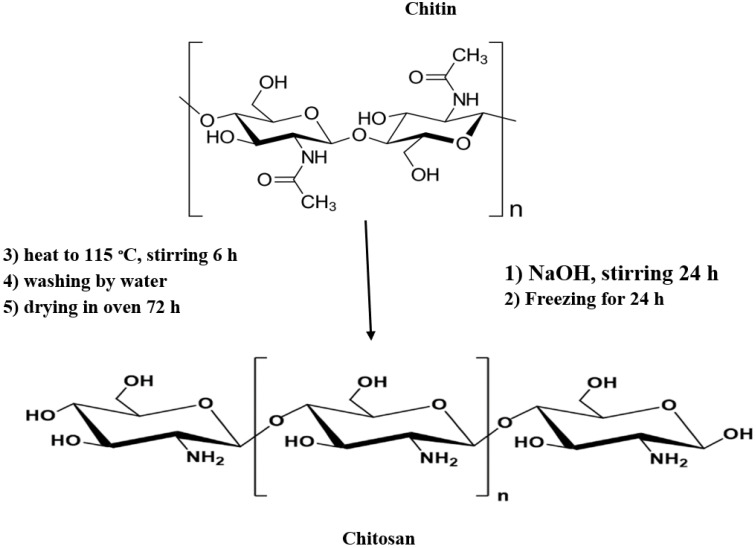
Preparation of chitosan from chitin.

The preparation of chitosan in this work was achieved by deacetylation of chitin, which was supplied from the shrimp peel as shown in Scheme 1. The chitin reacted with 12.5 M NaOH at a solid/liquid ratio of 1:15 (g/mL) and was stirred for 24 h. The mixture was cooled and frozen for 24 h. After that, the mixture’s temperature was raised to 115 °C, and the reaction continued while being stirred for 6 h. The crude product of chitosan was filtrated followed by washing with distilled water until neutral pH. Finally, the produced chitosan was dried at 70 °C [9].

### 2.2. Synthesis of Silver Nanoparticles (SNPs)

Using trisodium citrate to reduce AgNO_3_, an SNP colloidal solution was created. The process was carried out as follows: the solution was stirred for 30 min while adding 5 mL of 1% trisodium citrate dropwise after heating 50 mL of 1 × 10^−3^ M AgNO_3_ until boiling. This produced a pale-yellow color of SNPs [8].

### 2.3. Nano Formation of Chitosan with SNPs

The nano formation of the chitosan with SNPs (Chit-SNPs) was produced as follows: acetic acid solution of chitosan (5 mL) was added to 20 mL of SNPs aqueous solution. The mixed solution of chitosan and SNPs was stirred until the color changed to colorless [10].

### 2.4. Fabrication of the Nanocomposite (Chit-CNTs-SNPs)

The nanocomposite (Chit-CNTs-SNPs) was prepared using CNTs and Chit-SNPs as follows: the ethanolic solution of CNTs (1 g/100 mL) was poured into 50 mL solution of Chit-SNPs prepared in step (2.3) and was stirred for 24 h. The output material was filtered, and water-cleaned. The pure nanocomposite (Chit-CNTs-SNPs) was created after overnight drying under vacuum [11].

### 2.5. Removal of Ni (II) Ions Using the Nanocomposite (Chit-CNTs-SNPs)

The removal of Ni (II) ions using the nanocomposite (Chit-CNTs-SNPs) was carried out by the following steps.

#### 2.5.1. Preparation of Ni (II) Solutions

The nickel (II) sulfate hexahydrate (100 mg) was dissolved in 1 L of distilled water to prepare Ni (II) solution (100 mg/L) [3].

#### 2.5.2. Effect of Time on Removal of Ni Ions

In this work, Ni (II) solution (100 mg/L) and 0.1 g of adsorbent-prepared nanocomposite (Chit-CNTs-SNPs) at various periods (20, 40, 60, 80, 100, 120, 140, and 160 min) with pH 7 at ambient temperature were used to investigate the influence of time on the removal of Ni ions. To bring the adsorbent nanocomposite and the Ni ions into equilibrium, the solution was agitated for 90 min. A UV-VIS double-beam PC scanning spectrophotometer (LABOMED, INC., UV-2950, Los Angeles, CA, USA) was used to evaluate the solid portion of the filtered Ni (II) solutions as well as the leftover filtrates [3].

#### 2.5.3. Effect of pH on Removal of Ni Ions

A total of 0.1 g of the adsorbent (Chit-CNTs-SNPs) was combined with a series of Ni (II) solutions (100 mg/L). Using a standard acid solution of 0.01 M H_2_SO_4_ and an alkaline solution of 0.125 M NaOH, the pH of the solution was altered to be at pH levels of 1, 2, 10, and 11. Before taking any measurements, the pH meter was calibrated using buffers with pH values of 4.0, 7.0, and 10. The pH meter (Mettler-Toledo AG 8603 made by Mettler-Toledo Group, Schwerzenbach, Switzerland) was used to measure all pH measurements. To remove the solid component, the solutions were filtered. Then, using a UV-VIS double-beam PC scanning spectrophotometer (LABOMED, INC., UV-2950, Los Angeles, CA, USA), the residual Ni (II) concentrations were found [3].

#### 2.5.4. Nanocomposite Mass Effect on Sorption of Ni Ions

The mass effect of the nanocomposite as adsorbent for Ni ions was investigated by mixing of different weights (0.1, 0.2, 0.3, and 0.4 g) of the nanocomposite (Chit-CNTs-SNPs) separately to a series of 100 mL of Ni solutions (100 mg/L) at pH 7.0 while stirring for 90 min to reach the equilibrium. The solutions were filtered to separate the solid part of the nanocomposite. The remaining Ni (II) concentrations were detected using UV–VIS double-beam PC scanning spectrophotometer (LABOMED, INC., UV-2950, USA). We used Equations (1,2) to calculate the amount of adsorption qe (mg/g) and the percentage of removal (% removal) [3].

q_e_ = (C_0_ − C_e_) × V/m (1)

% removal = (C_0_ − C_e_)/C_0_ × 100% (2)

where C_0_ and C_e_ are the initial Ni (II) concentration and the concentration at equilibrium in mg/L, m is the mass of the adsorbent, and V is the volume of solution.

## 3. Experimental Analyses

### 3.1. Infrared Spectrometer (FTIR) and Ultraviolet Absorption Measurements (UV)

The FTIR measurements for the samples used in this study were carried using Thermos Nicolet 6700 FT-IR optical spectrometer (Mundelein, IL 60060 USA). The sample (2 mg) was grinded with 100 mg of KBr with grinding. The sample was converted to pellets by pressing into pills with a compressor. The wavenumber range of the spectra used was 4000–400 cm^−1^, with resolution of 4.0 cm^−1^ at an angle of incidence 80° relative to the surface normal. In this work, we used a UV–VIS double-beam PC scanning spectrophotometer (Labomed, Inc., UV-2950, Los Angeles, CA, USA). The distilled water was used as solvent for all measurements.

### 3.2. Scanning Electron Microscope, Energy-Dispersive X-Ray Spectroscopy, and Transmission Electron Microscope (TEM)

A VEGA 3 TESCAN scanning electron microscope (Tescan, Brno, Czech Republic) with a secondary electron (SE) detector and an energy-dispersive spectrometer (EDS) detector, Element Silicon Drift Detector AMETEK MATERIALS ANALYSIS DIVISION, USA, alongside an electron backscatter diffraction (EBSD) system, was employed for “in situ” chemical composition analysis, morphology observation, and structure determination of the prepared nanocomposite (Chit-CNTs-SNPs), where the measurement was carried out at a selected voltage of 20 KV with a working distance of 8–10 mm between the specimen and the detector without any polishing or use of conductive coating. The compositional characterization diffraction mapping of the nanocomposite (Chit-CNTs-SNPs) was carried out using EDX analysis. An investigation of the chitosan-SNPs nanostructure was conducted using TEM. The TEM images were measured by TEM model (Joel JeM–2100, Tokyo, Japan).

### 3.3. X-Ray Diffraction (XRD) and X-Ray Photoelectronic Spectroscopy (XPS)

The morphology composition of the fabricated nanocomposite (Chit-CNTs-SNPs) was investigated using X-ray diffraction (XRD-7000S, Shimadzu, Japan) operating with a Cu anticathode (λ = 1.5406 Å) at 40 kV and 30 mA, using a continuous scan mode of 2°/min with 2(θ) range from 20° to 100°. The XPS measurements for the nanocomposite (Chit-CNTs-SNPs) were performed using X-ray photoelectron spectroscopy, K-Alpha Plus, Thermo Fisher Scientific, Waltham, MA, USA, equipped with Al Kα multi-focused monochromatic.

### 3.4. Inductively Coupled Plasma Emission (ICPE) and Atomic Adsorption

The residual amount of Ni ions in nickel sulfate hexahydrate solution (100 mg/L) after the removal of Ni (II) from this solution using the nanocomposite (Chit-CNTs-SNPs) was determined using inductively coupled plasma emission spectroscopy (ICPE-9800, Shimadzu, Japan). In this work, the Perkin Elmer Analyst AA400 model flame atomic adsorption spectrometer (FAAS) (PerkinElmer Co., Norwalk, CT, USA) was used to determine the uptake of the Ni (II) ions absorbed by the nanocomposite (Chit-CNTs-SNPs).

## 4. Results and Discussion

### 4.1. Chemical Structure of the Synthesized Chitosan

The chemical structure of the synthesized chitosan in Scheme 1 was established using FTIR.

**Figure 1:**
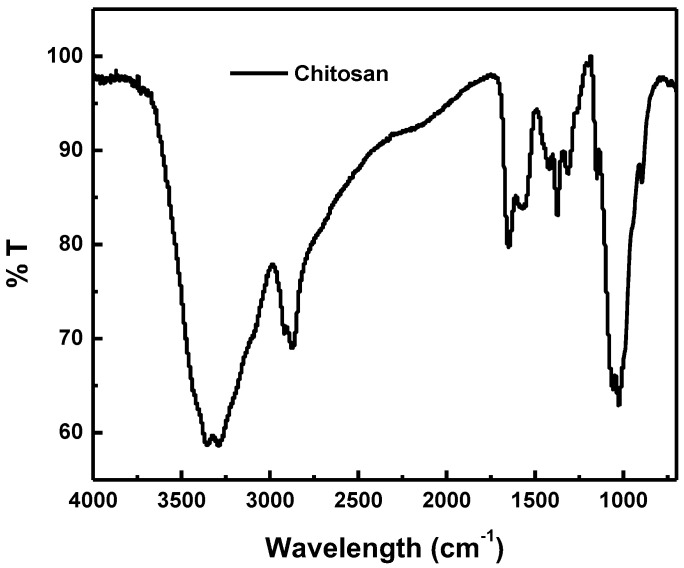
FTIR of the prepared chitosan.

We can observe the FTIR of the synthesized chitosan as shown in Figure 1. The bands in the region 3289–3362 cm^−1^ are related to the N–H and O–H stretching, as well as the intramolecular hydrogen bonds. These bands appear at 3291–3361 cm^−1^ as mentioned in a previous publication [12]. The bands that appear at around 2931 and 2876 cm^−1^ are the bands of C–H symmetric and asymmetric stretching, respectively, which are compatible with the position of these bands at 2921 and 2877 cm^−1^ mentioned in a previous work [12]. It is known that these bands are a typical characteristic of polysaccharides [12]. In addition, the band at 1575 cm^−1^ corresponds to the N–H bending of the primary amine [12]. The observed band at 1163 cm^−1^ can be considered as the asymmetric stretching of the C–O–C bridge. Moreover, the bands at 1071 and 1016 cm^−1^ correspond to C–O stretching [12]. The bands observed at 1648.79 and 1374.36 cm^−1^ show the presence of C=O stretching and C-H deformation, respectively [13]. All above bands are related to the spectra of chitosan as shown in other publications [12,13].

### 4.2. Chitosan Assembling on Silver Nanoparticles (SNPs)

**Figure 2:**
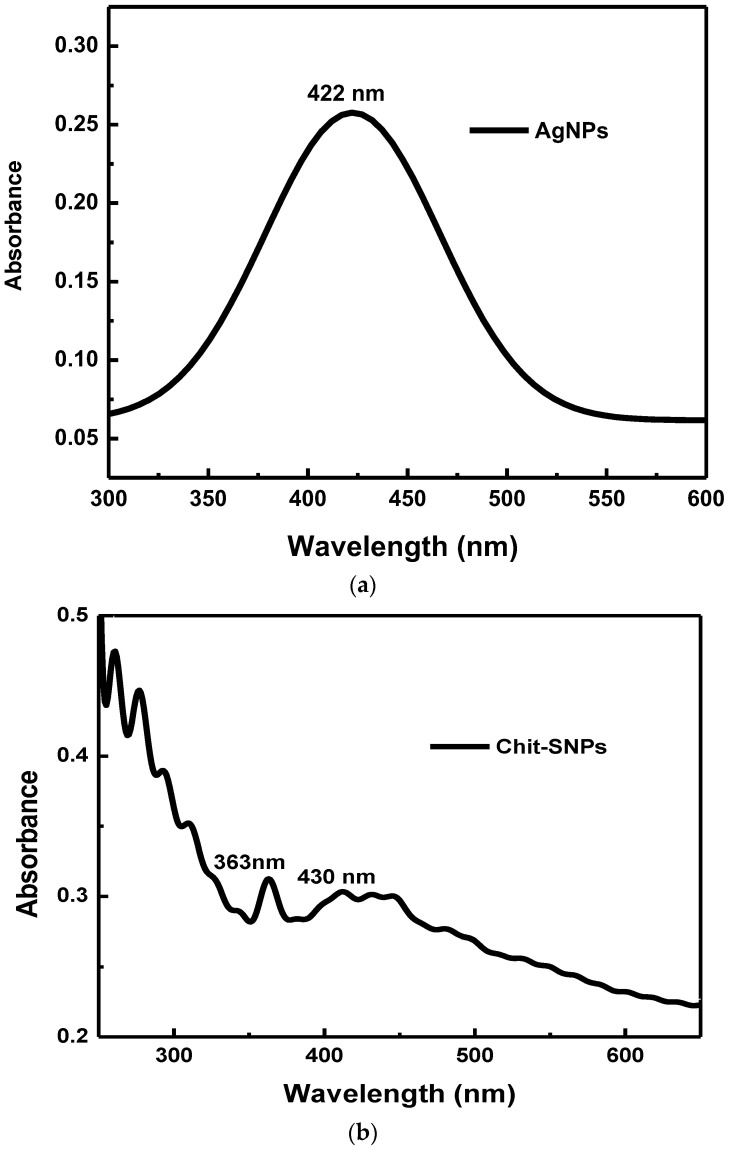
The UV-VIS spectra of SNPs (**a**) and Chit-SNPs (**b**).

**Figure 3:**
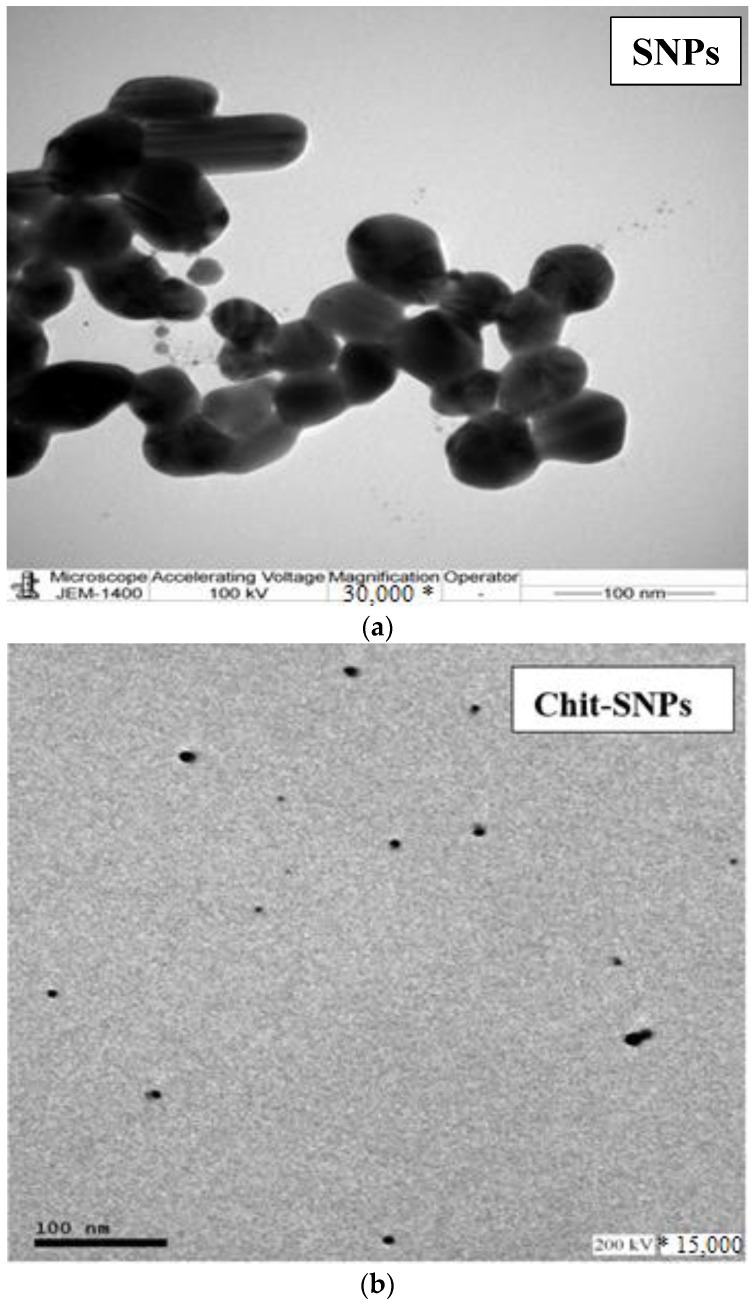
The TEM images of SNPs (**a**) and Chit-SNPs (**b**).

The assembling of chitosan molecules on SNPs was confirmed using UV absorbance spectrum and TEM as shown in Figure 2 and Figure 3. Figure 2 shows the absorbance spectra of SNPs individually and after the assembling of chitosan molecules. The absorbance peak for SNPs appears at 422 nm because of the surface plasmon absorption of SNPs. This peak disappears after the assembling of chitosan molecules on SNPs as shown in Figure 3. The reason for this phenomenon is related to the neutralization of the charges surrounding the SNPs and reduction in the plasmon charge on it [14]. Further confirmation of the assembling of chitosan molecules on SNPs was achieved using TEM images as shown in Figure 3. TEM micrographs of the individual SNPs solution and the SNPs in the presence of chitosan molecules are represented in Figure 3. The nanostructure of SNPs in Figure 3 can be seen as taking a spherical shape and polycrystalline structure, with an average size range from 19.1 to 21.44 nm. After assembling chitosan molecules on the SNPs, the individual nanoparticles converted to nanoshells. This behavior leads to restricting the aggregation of SNPs due to the formation of nanoshells after the assembling of chitosan molecules into SNPs (see Figure 3). Moreover, the assembling of chitosan molecules increased the stability of the SNPs and reduced their aggregation. Therefore, the presence of NH_2_ groups in chitosan enhances the assembling of the chitosan molecules on SNPs via chemosorption due to the presence of a lone pair of electrons on the N_2_ atom in NH_2_ groups. In addition, due to the effect of NH_2_ groups in chitosan molecules on the charge of SNPs, the surface charge of the SNPs is reduced [15]. The above factors prevented the aggregation of SNPs after the formation of nanoshells with chitosan molecules as shown in Figure 3.

### 4.3. Establishment of the Morphology and Composition of the Prepared Nanocomposite (Chit-SNPs-CNTs)

The morphology and composition of the prepared nanocomposite (Chit-SNPs-CNTs) was studied using some techniques as follows.

#### 4.3.1. FTIR Spectroscopy of the (Chit-SNPs-CNTs)

**Figure 4:**
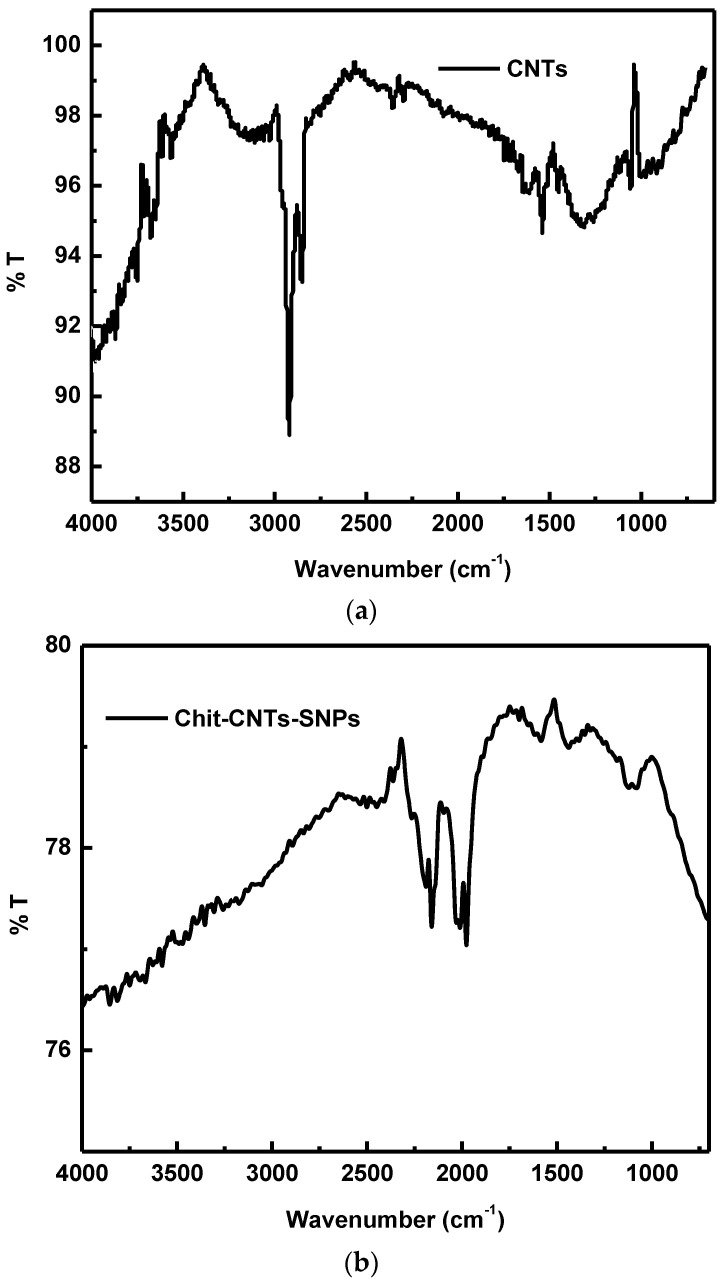
The FTIR spectra of the CNTs and Chit-CNTs-SNPs nanocomposite.

Figure 4 represents the FTIR spectra of the CNTs and Chit-CNTs-SNPs. We used the FTIR to confirm the formation of the Chit-CNTs-SNPs nanocomposite by testing the vibration frequency changes in their functional groups within the range of 4000–400 cm^−1^. The FTIR of the individual CNTs showed absorption bands at 2860 and 2917 cm^−1^ for symmetric/asymmetric CH_2_ groups stretching, respectively. These groups may be located at the defect sites on the sidewall surface [16]. After the formation of the nanocomposite (Chit-CNTs-SNPs), the FTIR of CNTs in Figure 4 is completely changed and new bands appear at 2162, 2015, and 1979 cm^−1^, which are related to the stretching vibration of SNPs and the CN group of chitosan, respectively [17].

#### 4.3.2. Energy-Dispersive X-ray (EDX)

**Figure 5:**
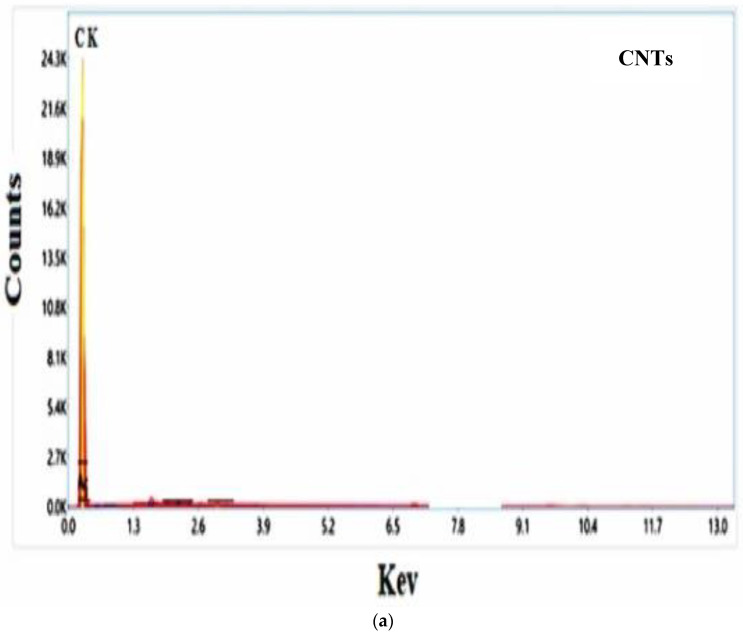

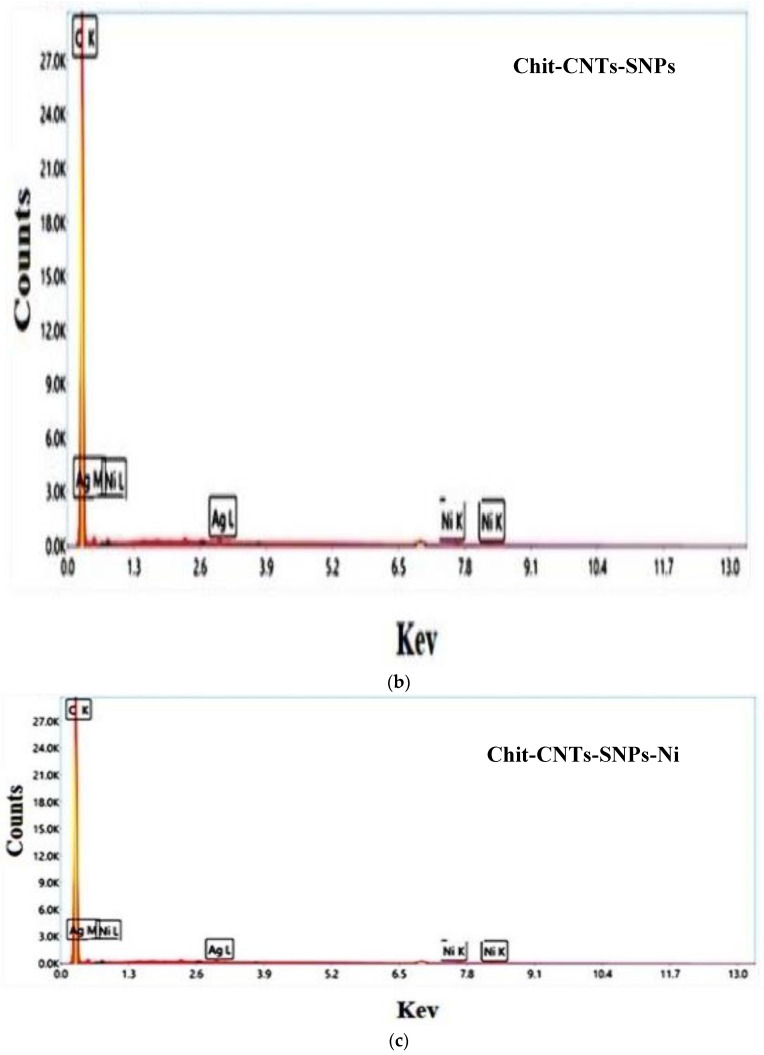
THE EDX of CNTs (**a**), Chit-CNTs-SNPs (**b**), and Chit-CNTs-SNPs-Ni (**c**).

The EDX chart in Figure 5 for CNTs represents the C (K) peak for the CNTs. After the addition of SNPs coated with chitosan, new peaks for Ag (M) and Ag (L) appeared with the C (K) peak of CNTs as shown in Figure 5, which gives an indication of the formation of the (Chit-CNTs-SNPs) nanocomposite.

#### 4.3.3. Scanning Electron Microscope (SEM)

**Figure 6:**
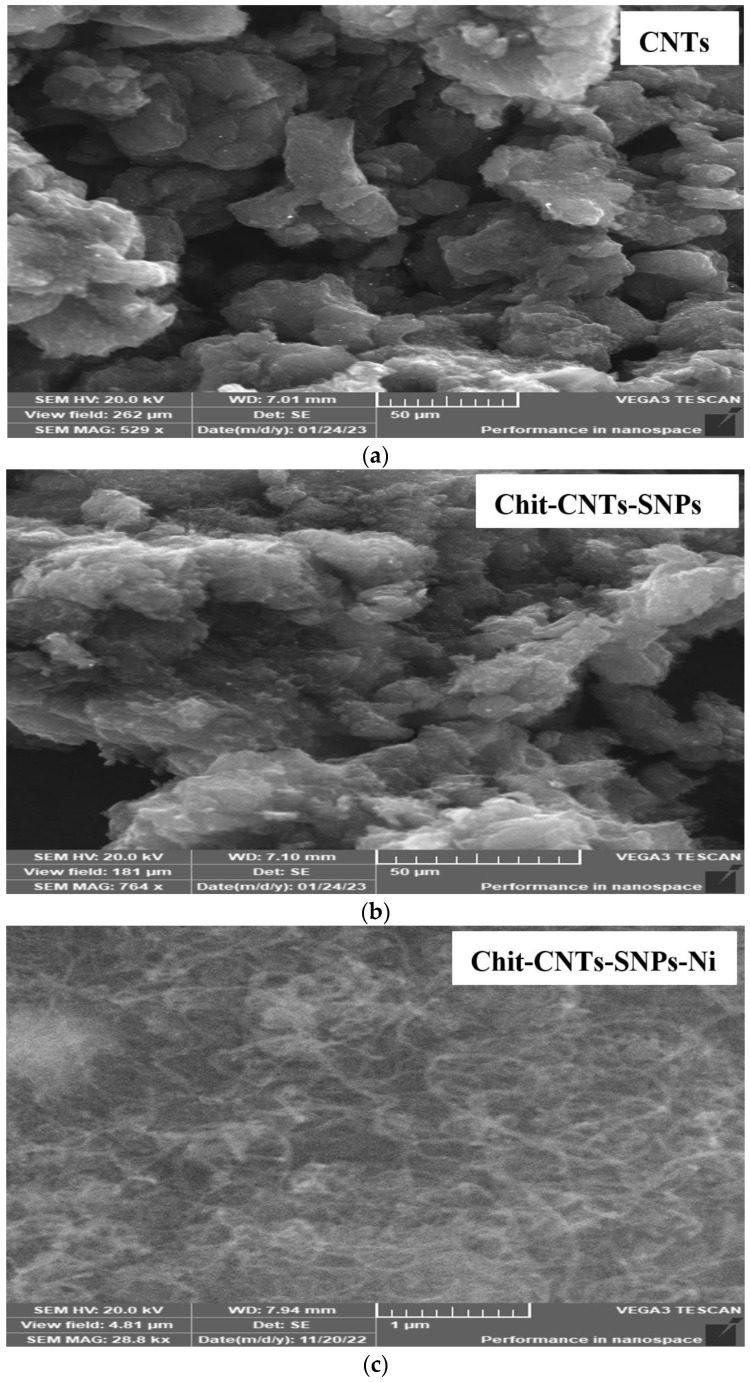
SEM images of CNTs (**a**), Chit-CNTs-SNPs (**b**), and Chit-CNTs-SNPs-Ni (**c**).

In this study, we used an SEM to study the surface morphology of the CNTs and the prepared nanocomposite (Chit-CNTs-SNPs) as shown in Figure 6. It can be noticed from the SEM images of the CNTs in Figure 6 that the surface of the CNTs contains smooth and wide blanks between the layers. It is clear from Figure 6 that the blanks between the CNTs layers decreased after the addition of the SNPs coated with chitosan. This may be due to the filling of these blanks by the SNPs coated by the chitosan molecules in the prepared nanocomposite (Chit-CNTs-SNPs) [6].

#### 4.3.4. X-ray Diffraction (XRD)

**Figure 7:**
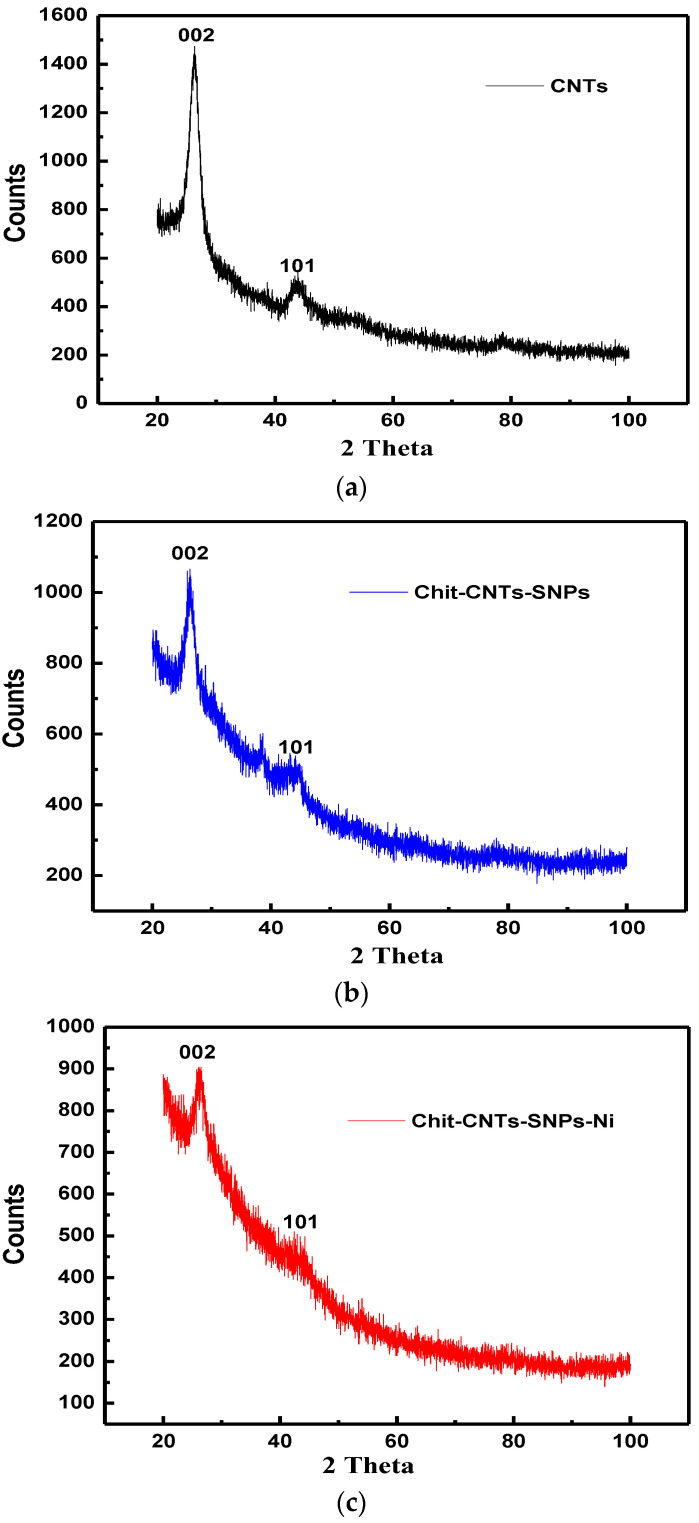
XRD of CNTs (**a**), Chit-CNTs-SNPs (**b**), and Chit-CNTs-SNPs-Ni (**c**).

In addition to the SEM and EDX, we used XRD for a further study of the morphology of the fabricated nanocomposite (Chit-CNTs-SNPs) as represented in Figure 7. The XRD pattern CNTs show a (002) peak at ∼26.3° and (101) peak at 43.7°. These peaks arise due to the tubular structure of the carbon atoms [18]. The intensity of this peak decreased after the addition of SNPs coated by chitosan molecules to form the nanocomposite (Chit-CNTs-SNPs) as shown in Figure 7. This may be due to the interpenetration of the SNPs coated by chitosan between the CNTs layers, which confirms the formation of the fabricated nanocomposite (Chit-CNTs-SNPs).

#### 4.3.5. X-ray Photoelectronic Spectroscopy (XPS)

**Figure 8:**
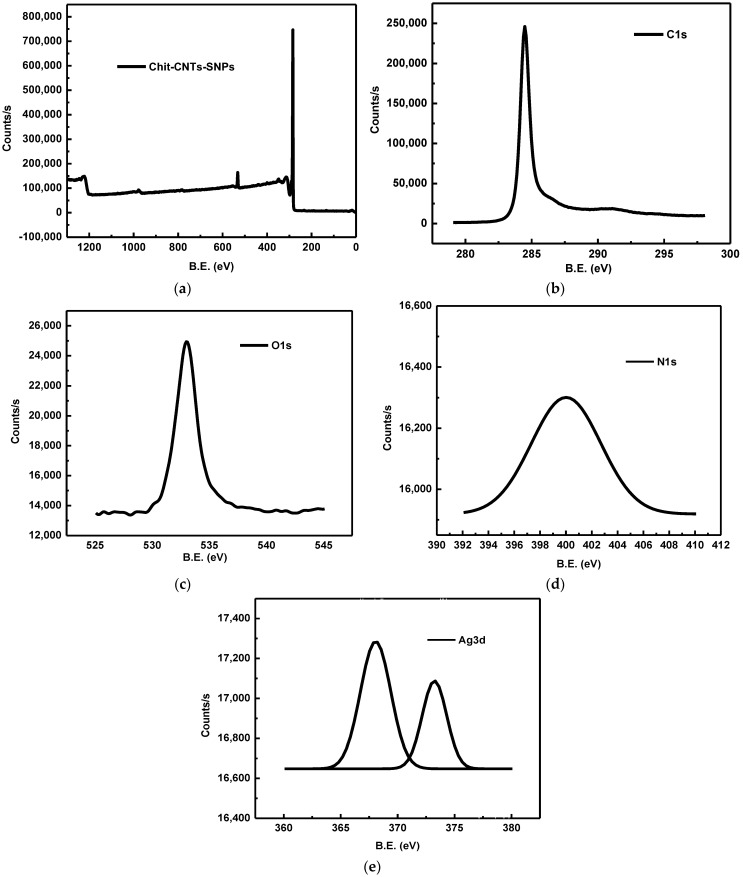
XPS spectra of Chit-CNTs-SNPs nanocomposite. (**a**) Chit-CNTs-SNPs; (**b**) C1s; (**c**) O1s; (**d**) N1s; (**e**) Ag3d.

The composition of the fabricated nanocomposite (Chit-CNTs-SNPs) was also confirmed using the XPS technique. Figure 8 represents XPS spectra of the nanocomposite (Chit-CNTs-SNPs). When focused into the region characteristic for the carbon, the C1s peak of the CNTs was resolved at 284.5 eV and the O1s peak of O_2_ was detected at 532.2 eV as shown in Figure 8 [19]. After the addition of SNPs coated with chitosan molecules to the CNTs for the formation of the nanocomposite (Chit-CNTs-SNPs), new peaks appeared as represented in Figure 8. One peak appeared at 400.54 eV (N 1s) for N_2_ atoms, and two peaks appeared at 368.02 (Ag3d 5/2) and 373.23 eV (Ag3d 3/2) with respect to the silver nanoparticles (SNPs).

### 4.4. The Uptake of Ni (II) Ions from Nickel Sulphate Solution

**Figure 9:**
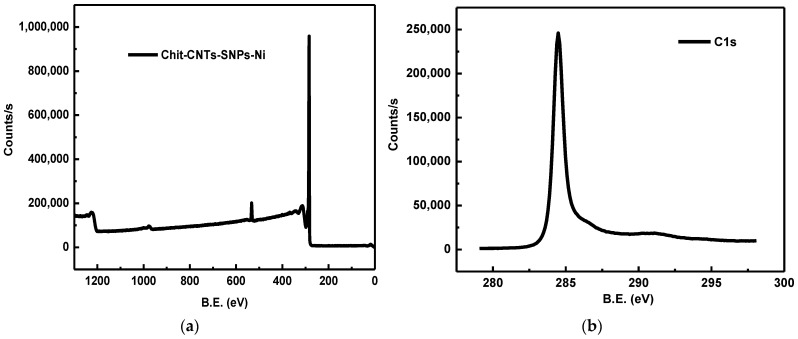

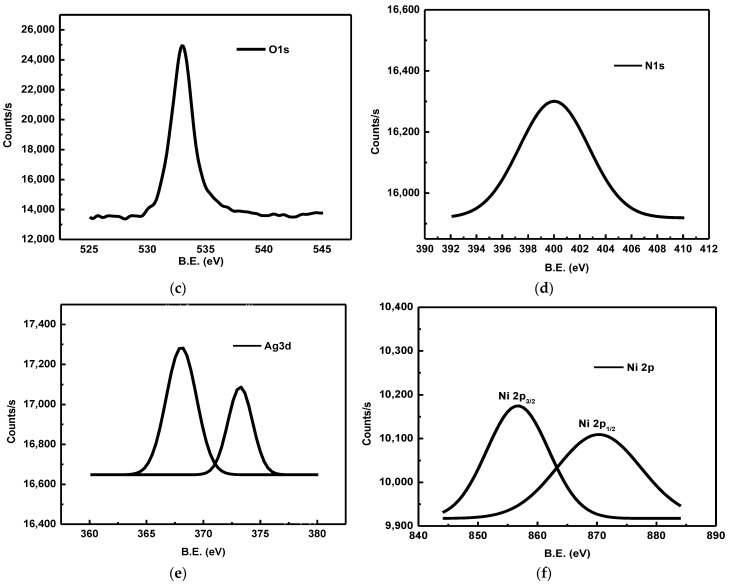
XPS spectra of Chit-CNTs-SNPs-Ni. (**a**) Chit-CNTs-SNPs-Ni; (**b**) C1s; (**c**) O1s; (**d**) N1s; (**e**) Ag3d; (**f**) Ni2p.

The following techniques were used in this work to establish the uptake of Ni (II) ions from the nickel sulfate hexahydrate (NiSO_4_·6H_2_O) solution onto the fabricated nanocomposite (Chit-CNTs-SNPs): the EDX results in Figure 5 were used to confirm the removal of Ni (II) ions using the fabricated nanocomposite (Chit-CNTs-SNPs). It is clear in Figure 5 that some peaks related to Ni (L) and Ni (K), in addition to the other peaks for Ag (M) and Ag (L), appeared with the C (K). The presence of the Ni peaks in Figure 5 establishes the sorption of Ni ions onto the surface of the nanocomposite (Chit-CNTs-SNPs). The SEM image in Figure 6 shows the surface morphology of the nanocomposite after the sorption of Ni ions (Chit-CNTs-SNPs-Ni). It is clear from this image that the surface morphology is completely changed after the uptake of Ni (II) onto the surface of the Chit-CNTs-SNPs nanocomposite. The surface contains bumps, small dark gray spots, and tight thickness due to the uptake of Ni (II) ions. Herein, we used XRD in this work for the investigation of the sorption of Ni (II) onto the fabricated nanocomposite (Chit-CNTs-SNPs) as shown in Figure 7. As mentioned in Figure 7, the XRD pattern CNTs show a (002) peak at ∼26.3° and (101) peak at 43.7°. The intensity of these peaks of CNTs became weaker, as shown in Figure 7, which may be due to the sorption of Ni ions onto the surface of the Chit-CNTs-SNPs nanocomposite. XPS analysis was used here to gain further investigation into the sorption of Ni (II) ions as shown in Figure 8. Comparing between the XPS of Chit-CNTs-SNPs (see Figure 8) and the XPS after sorption of Ni in Figure 9, it is noticed that new peaks appear at binding energy (BE) values of 856.6 and 870.2 eV related to Ni2p, which consists of two spin-orbit doublets, Ni2p_3/2_ and Ni2p_1/2_, respectively [20]. These results confirm the uptake of the Ni ions on the surface of the Chit-CNTs-SNPs nanocomposite. The uptake of Ni (II) using 0.1 g of the prepared nanocomposite (Chit-CNTs-SNPs) was investigated in this work using ICPE and atomic adsorption as represented in Table 1. The result of atomic adsorption shows that 72.70 ppm of Ni (II) ions were uptaken from a 100 ppm NiSO_4_.6H_2_O solution. In addition, the ICPE data in Table 1 confirm that 27.30 ppm of Ni (II) ions remain in the NiSO_4_.6H_2_O solution. These results in Table 1 confirm the uptake of Ni (II) ions using the Chit-CNTs-SNPs nanocomposite.

Moreover, the uptake of Ni (II) ions using the fabricated nanocomposite (Chit-CNTs-SNPs) was investigated experimentally using the UV-VIS technique at different contact times, pHs of the Ni (II) solution, and masses of the adsorbent (Chit-CNTs-SNPs) nanocomposite as follows.

### 4.5. Variation in Contact Time

**Figure 10:**
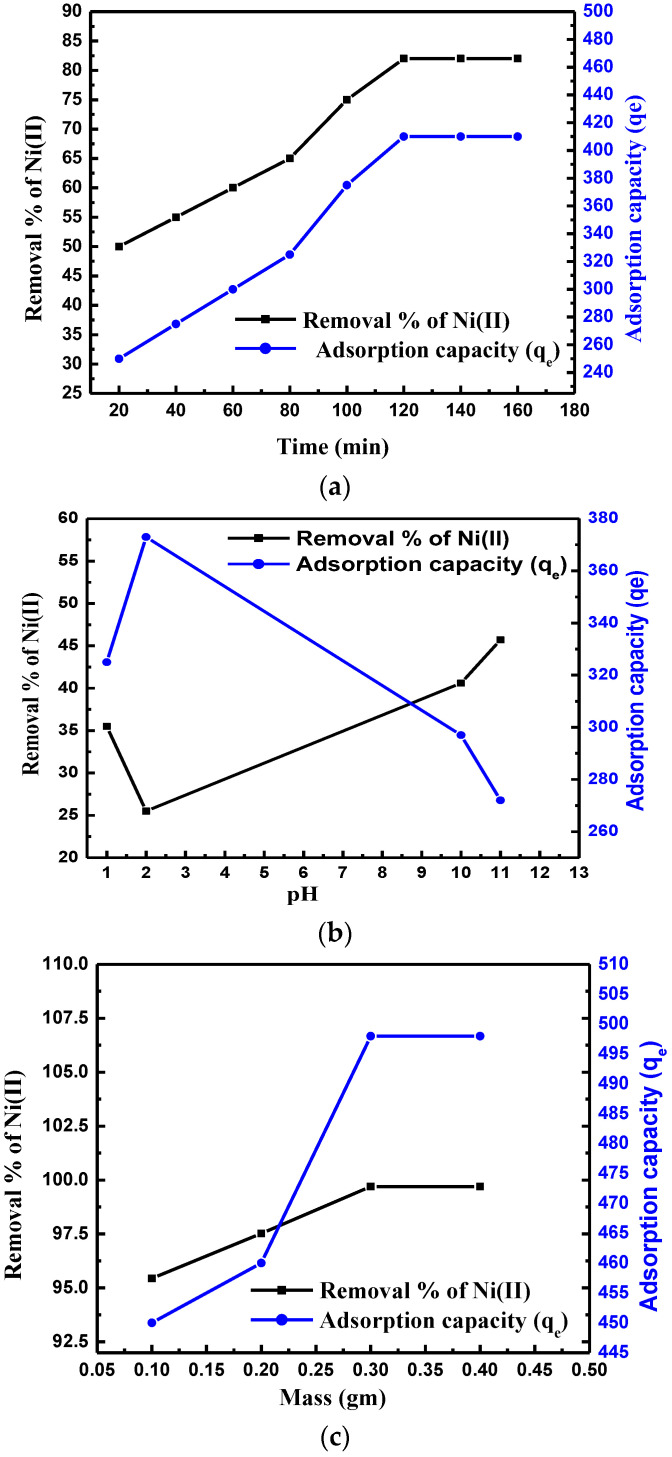
Effect of contact time (**a**), pH (**b**), and the mass of nanocomposite (Chit-CNTs-SNPs) (**c**) on the sorption Ni ions.

The data in Figure 10 and Table 1 represent the relationship between the contact time and the absorbent (Chit-CNTs-SNPs) nanocomposite’s adsorption capacity (qe). The contact time varied at 20, 40, 60, 80, 100, 120, 140, and 160 min, with an initial metal concentration of nickel (II) sulfate hexahydrate of 100 mg/L. The analysis of the data in Figure 10 and Table 1 shows that the adsorption rate of Ni (II) ions using the nanocomposite (Chit-CNTs-SNPs) increases from a 50% removal of Ni (II) and q_e_ = 250 mg of Ni (II) per gram of (Chit-CNTs-SNPs) at 20 min until 82% and q_e_ = 410 mg of Ni (II) per gram of (Chit-CNTs-SNPs) at 120 min. These results in Figure 10 and Table 1 make it abundantly evident that the chelation between the Ni ions and the NH_2_ group in chitosan may play a role in the adsorption of Ni (II) using the (Chit-CNTs-SNPs) nanocomposite. The rapid diffusion of Ni (II) ions from the solution to the outer surface (Chit-CNTs-SNPs) as a result of the interaction between CNTs and SNPs in the nanocomposite (Chit-CNTs-SNPs) may also be connected to this [3].

### 4.6. Variation in pH

It is known from the previous publications that the variation in pH of the metal ion solution has an effect on the adsorption capacity (q_e_) of an absorbent material such as the (Chit-CNTs-SNPs) nanocomposite [3]. Figure 10 and Table 2 show the results of the investigation into how the pH of the starting solution affects the removal percentage of Ni (II) and adsorption capacity of the synthesized nanocomposite (Chit-CNTs-SNPs). The high percentage (45.70%) of the Ni (II) ions adsorbed by the (Chit-CNTs-SNPs) nanocomposite was found at pH = 11 as represented in Figure 10 and Table 2. The NH_2_ groups of chitosan in the nanocomposite (Chit-CNTs-SNPs) convert to –NH^3+^ cation at a lower pH (1 and 2), which deactivates the chelation between the NH_2_ group and Ni (II) ions. Moreover, the conversion of the NH_2_ groups to the –NH^3+^ cation causes electrostatic repulsion between Ni (II) ions and –NH^3+^ cation, which restricts the sorption of Ni (II) ions using the nanocomposite (Chit-CNTs-SNPs) [3]. At a high pH (10 and 11), the Ni (II) starts to deposit as Ni(OH)_2_, which causes a decrease in the adsorption capacity (q_e_ = 297 at pH = 10 and 272 at pH = 11) as shown in Figure 10 and Table 2 [3].

### 4.7. Variation in the Nanocomposite Mass

The removal % of Ni (II) and the adsorption capacity were considered in this work by a mass variation in the fabricated nanocomposite (Chit-CNTs-SNPs) from 0.1 to 0.4 g at a constant pH and contact time as shown in Table 3 and Figure 10. It is clear from the data in Table 3 and Figure 10 that the maximum removal of Ni (II) ions reaches (99.70%) and the adsorption capacity (qe) equals (498 mg/g) using 0.3 g of the (Chit-CNTs-SNPs) nanocomposite. Comparing these data in Table 4 and Figure 10 with the results given in the previous publications [3] gives an indication that the sorption of Ni (II) ions using the fabricated nanocomposite (Chit-CNTs-SNPs) in this work has a greater efficiency for the removal of heavy metal ions than the other composites in the previous publications.

### 4.8. The Mechanism for the Uptake Process of Ni (II) Ions

The fabrication of the nanocomposite (Chit-CNTs-SNPs), which is used to adsorb Ni (II) ions, depends on a number of variables. One of these elements is the coordination between the metal cation and amino group in chitosan, which causes chelation [6,21]. The amino groups of the chitosan molecule, which function as a Lewis base, can donate electron pairs to the metal cation, such as Ni (II), which acts as a Lewis acid [3]. As the nanocomposite (Chit-CNTs-SNPs) contains ions exchangeable with silver nanoparticles (SNPs) in addition to the metal chelation with the NH_2_ group of chitosan, the removal of the Ni (II) ions in this work depends on both the ion-exchange mechanism and the chelation [3,21]. The information in Table 4 demonstrates that the synthesized nanocomposite (Chit-CNTs-SNPs), which was used as an absorbent in this study, was more effective in absorbing heavy metal ions than other absorbents in earlier studies [3].

## 5. Conclusions

Recently, greater focus has been placed on creating environmentally acceptable nanocomposites that function as adsorbents, particularly those that rely on biopolymers, CNTs, and nanoparticles. Here, we created an environmentally friendly nanocomposite (Chit-CNTs-SNPs) for absorbing Ni (II). We looked into how the mass of the absorbent, pH of the solution, and contact time affected the removal of Ni (II) ions. According to the results of this work, 0.3 g of the Chit-CNTs-SNPs nanocomposite had the highest removal rate (99.70%) of Ni (II). The results also show that qe = 498 mg/g was the critical value for this work’s maximal Ni (II) ion uptake. The Chit-CNTs-SNPs nanocomposite is also very active in capturing Ni (II) ions from wastewater treatment.

## Figures and Tables

**Table 1:** Removal % of Ni II ions adsorption capacity (q_e_) using UV absorbance at different times using the fabricated nanocomposite (Chit-SNPs-CNTs) at pH = 7 and ambient temperature.

Time (min)	20 min	40 min	60 min	80 min	100 min	120 min	140 min	160 min
Removal %	50%	55%	60%	65%	75%	82%	82%	82%
C_e_ (g/l)	0.050	0.045	0.040	0.035	0.025	0.018	0.018	0.018
q_e_ (mg/g)	250	275	300	325	375	410	410	410

**Table 2:** Removal % of Ni II ions adsorption capacity (q_e_) using UV absorbance at different pH using the fabricated nanocomposite (Chit -CNTs-SNPs) after 1 h at ambient temperature.

pH of Ni (II) Solution	pH = 1	pH = 2	pH = 10	pH = 11
Removal %	35.50%	25.50%	40.60%	45.70%
Ce (g/l)	0.035	0.0255	0.0406	0.0457
qe (mg/g)	325	373	297	272

**Table 3:** Removal % of Ni (II) ions and adsorption capacity (q_e_) using UV absorbance at different weights using the fabricated nanocomposite (Chit-CNTs-SNPs) after 1 h at pH = 7 and ambient temperature.

Mass of (Chit-CNTs-SNPs)	0.1 gm	0.2 gm	0.3 gm	0.4 gm
Removal %	95.44%	97.53%	99.70%	99.70%
C_e_ (g/l)	0.0955	0.0080	0.0003	0.0003
q_e_ (mg/g)	450	460	498	498

**Table 4:** Comparison between the maximum adsorption capacity (mg/g) of the fabricated nanocomposite (Chit-CNTs-SNPs) and some absorbents for heavy metals removal.

Absorbent	Maximum Adsorption Capacity (mg/g)	Reference
Chitosan–zeolite composites	14.75–51.32	3
Semi-IPN hydrogel based on chitosan and gelatin	153.9	3
Xanthate-modified magnetic chitosan	34.5	3
Chitosan nanofibrils	186.6	3
Chitosan (Ch)/clay, chitosan (Ch)/AgNPs/clay, and chitosan (Ch)/AuNPs/clay	181.5	3
Chit-CNts-SNPs	498	Present work

## Data Availability

The data presented in this study are available on request from the corresponding author. The data are not publicly available due to privacy.
